# Patterns of genetic diversity in southern and southeastern *Araucaria angustifolia* (Bert.) O. Kuntze relict populations

**DOI:** 10.1590/S1415-47572009005000052

**Published:** 2009-09-01

**Authors:** Maria Isabel Ferreira de Souza, Fabiano Salgueiro, Mariana Carnavale-Bottino, Durvalina Benedita Félix, Marcio Alves-Ferreira, Juliana Vitoria Messias Bittencourt, Rogério Margis

**Affiliations:** Laboratório de Genética Molecular Vegetal, Departamento de Genética, Instituto de Biologia, Universidade Federal do Rio de Janeiro, Rio de Janeiro, RJBrazil; 2 Departamento de Genética, Instituto de Biologia, Universidade Federal Rural do Rio de Janeiro, Rio de Janeiro, RJBrazil; 3 Universidade Tecnológica Federal do Paraná, Campus Dois Vizinhos, Paraná, PRBrazil; 4 Laboratório de Genomas e Populações de Plantas, Centro de Biotecnologia, Universidade Federal do Rio Grande do Sul, Porto Alegre, RSBrazil; 5 Departamento de Bioquímica, Universidade Federal do Rio Grande do Sul, Porto Alegre, RSBrazil

**Keywords:** Araucariaceae, relict populations, araucaria forest, population genetics, AFLP

## Abstract

Habitat fragmentation and a decrease in population size may lead to a loss in population genetic diversity. For the first time, the reduction in genetic diversity in the northernmost limit of natural occurence (southeastern Brazil) of *Araucaria angustifolia* in comparison with populations in the main area of the species continuous natural distribution (southern Brazil), was tested. The 673 AFLPs markers revealed a high level of genetic diversity for the species (*Ht* = 0.27), despite anthropogenic influence throughout the last century, and a decrease of *H* in isolated populations of southeastern Brazil (*H* = 0.16), thereby indicating the tendency for higher genetic diversity in remnant populations of continuous forests in southern Brazil, when compared to natural isolated populations in the southeastern region. A strong differentiation among southern and southeastern populations was detected (AMOVA variance ranged from 10%-15%). From Bayesian analysis, it is suggested that the nine populations tested form five “genetic clusters” (*K* = 5). Five of these populations, located in the northernmost limit of distribution of the species, represent three “genetic clusters”. These results are in agreement with the pattern of geographic distribution of the studied populations.

## Introduction

Deforestation is one of the main factors leading to forest fragmentation, or in other words, the complete transformation of a large area from a continuum of habitats to a large number of small patches of vegetation isolated one from the other (Macedo, 1993; Young *et al.*, 1996). Habitat fragmentation may lead to a loss in population genetic diversity through different processes. In the first instance, a drastic reduction in population size can result in the loss of small frequency alleles. This usually occurs since part of the remanescent individuals may constitute an insignificant sample of the original genetic pool (Frankel and Soulé, 1981). It is impossible to predict to what extent this may occur. Thus, it is extremely important to be well-aware of both the levels of reduction in forest vegetation as well as the genetic structure of natural populations present during the fragmentation process. The immediate loss of heterozigosity will only be noticed in the event of the population suffering a large reduction in size (White *et al.*, 1999).

The fragmentation of forests into small patches is responsible for the diminishing habitat-area (Watts *et al.*, 2005), and for a reduction in the effective size of reproductive individuals, thereby increasing the probability of inbreeding among related trees. If this actually occurs, a consequential loss of genetic diversity is expected over long term, mainly due to stochastic events associated with inbreeding populations of diminished size and genetic drift (Menges, 1991; Ellstrand and Elam, 1993; Bouzat, 2001).

*Araucaria angustifolia* (Bertol.) Kuntze, also known as the Brazilian pine, is a dioecious wind-pollinated species whose seeds are dispersed mainly by authocory. *A. angustifolia* is one of the most important trees in its natural range of distribution, due to its economical, social and ecological relevance. As a result of the high quality of its timber, the wood is used for construction in general, furniture making and the production of long-fibre cellulose (Carvalho, 2003). Furthermore, through being rich in starch, the seeds constitute an important source of nutrients for humans (Reitz *et al.*, 1988). Until now, araucaria buds are used in popular medicine (Marquesini, 1995). As to the species ecological relevance, it is known by its typical pioneer behavior (Reitz *et al.*, 1988) and for being a sun-loving species during the very first stages of development (Rizzini, 1976). Hence, populations formed by adult individual araucaria make it possible for shade-tolerant plants of different taxa to grow and develop properly (Carvalho, 2003).

According to the records, the estimated natural area of distribution of *A. angustifolia* was formerly about 200,000 square kilometers (Reitz *et al.*, 1988). However, at the beginning of the last century, the species underwent indiscriminate exploitation, mainly as a result of its social and economic relevance (Guerra *et al.*, 2002), thus occasioning a severe reduction of this natural area. It is estimated that, at present, only about 1 to 5% of the former *A. angustifolia* natural range still remains, thus placing the species in the critically endangered category (IBAMA, 1992; IUCN, 2008).

**Figure 1:**
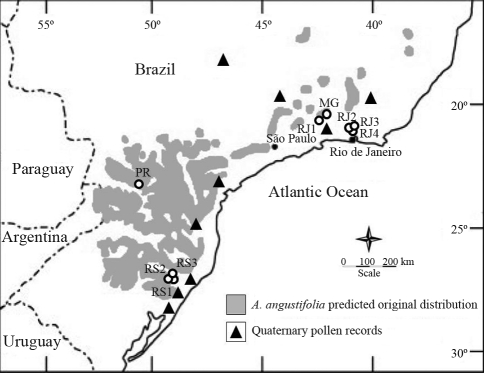
Map showing the estimated original distribution of *Araucaria angustifolia* in Brazil, location of late Quaternary pollen records containing *Araucaria* (Kershaw and Wagstaff, 2001) and the sampled populations: RS-1, RS-2, RS-3, PR, MG, RJ-1, RJ-2, RJ-3 and RJ-4.

Nowadays, araucaria forests are restricted to altitudes above 600 m, over a wide natural range in the three southernmost states of Brazil (Rio Grande do Sul, Santa Catarina and Paraná), between latitudes 24° and 30° S. The species is also sparsely spread throughout other states in Brazil, such as Minas Gerais, São Paulo and Rio de Janeiro, as isolated, relict populations, between latitudes 18 and 24° S and at higher altitudes (1200 m). It also occurs as a small extant population in the Province of Missiones, in Argentine (Hueck, 1972; Mattos, 1994) (Figure 1). However, in the past, the species was spread further north. Ruschi (1950) describes a no longer existent population of araucaria from the southern region of the state of Espírito Santo (Serra do Caparaó, Latitude 20° 26' S, 1700 m elevation). Based on palynological studies, Ledru *et al.* (1996) reported the presence of *A. angustifolia* pollen records from the Late Pleistocene in the “Lagoa Campestre” lake in Salitre, in the state of Minas Gerais (19° S, 46° 46' W, at 970 m). Studies based on the “Bioclim” algorithm (Busby, 1991), as mentioned by Koch *et al.* (2007), and that take into account data on species occurrence, mean pluviometry and mean temperature, confirm that araucaria forests can occur at lower latitudes.

It is possible that the depletion of wide areas of araucaria forests may have led to a decrease in genetic diversity, to the point of interfering in its use for conservation and exploitation of its genetic resources. At present, a large number of approaches have been undertaken by using various markers, all of which point to the fact that, notwithstanding araucaria forests having undergone drastic reduction in areas of natural distribution, a considerable level of genetic diversity has still been maintained (Shimizu *et al.*, 2000; Medri *et al.*, 2003; Mantovani *et al.*, 2006, Stefenon *et al.*, 2007). Nevertheless, most of the present studies have focused on remnant populations of continuous forests in southern Brazil. To date, there are no studies focusing on analyzing genetic diversity in natural, isolated and relict populations of *A. angustifolia* in southeastern Brazil. The main goal of the present study was to evaluate the reduction in genetic diversity of five *A. angustifolia* populations in southeastern Brazil, when compared with other populations from the south (the descendants of continuous forests).

## Methodology

###  Sampling

In order to analyse a large part of the natural range of *A. angustifolia*, so as to compare genetic diversity between remnants of continuous forests in southern Brazil and isolated populations in the southeastern region, nine different populations located in the main area of distribution and in the northernmost limit of distribution of the species in Brazil, were sampled. Individuals from four populations in southern Brazil - São Francisco de Paula, in the state of Rio Grande do Sul (RS-1, RS-2 and RS-3) and Mangueirinha, in the state of Paraná (PR) - and individuals from five populations in southeastern Brazil - Itatiaia, Rio de Janeiro (RJ-1), Teresópolis, Rio de Janeiro (RJ-2, RJ-3 and RJ-4) and Liberdade, in the state of Minas Gerais (MG) - were sampled, comprising a total of 190 individuals (Table 1). In Teresópolis, where only small isolated patches of araucaria are to be found, and even so, normally located in grassland and pastures, all available individuals were sampled. Populations from the states of Rio Grande do Sul and Paraná consisted of fragments of continuous forests. On the other hand, populations from Minas Gerais and Rio de Janeiro were naturaly isolated by climatic and physical conditions. In each population, individuals were collected from 1 ha plots, the sampled trees being at the minimum 15 cm in diameter at breast height (DBH). Needle and cambium samples were stored during the collecting period (1-7 days) in silica gel and later kept in a freezer at - 80 °C.

###  DNA extraction and AFLP analysis

Needles and cambium were extracted according to the CTAB method (Doyle and Doyle, 1990) with modifications (Margis *et al.*, 2002). AFLP analyses were perfomed based on Vos *et al.* (1995). Selective amplifications were done on the pre-amplified fragments by using six primer-enzyme combinations (PECs): *Eco*RI-CA/*Mse*I-GACC, *Eco*RI-CA/*Mse*I-GCAC, *Eco*RI-CC/*Mse*I-GACC, *Eco*RI-CG/*Mse*I-GACC, *Eco*RI-CG/*Mse*I-GCAC and *Eco*RI-CT/*Mse*I-GACC. The *Eco*RI primers were fluorescently labelled with either hexachlorofluorescein or 6-carboxyfluorescein (invitrogen©). A 3100 Genetic Analyzer ABI Prism 377 (Applied Biosystem®) automatic sequencer was used to obtain the electropherograms. Fluorescent AFLP patterns were scored with a Gene Mapper 3.5 (Applied Biosystem©). The AFLP profiles of each individual were scored for band presence (1) or absence (0) to create binary matrices. Population allele frequency data were estimated from AFLPs loci, assuming Hardy-Weinberg equilibrium as described by Lynch and Milligan (1994).

###  Data analysis

#### Genetic diversity

The percentage of polymorphic loci estimates were calculated for each primer-enzyme combination (PEC) and for the six PECs together, based on 99% criteria, and using Population Genetic Analysis (TFPGA) software (Miller, 1997).

Genetic diversity indices were computed among individuals within the nine populations. First we considered the three populations from Teresópolis (RJ-2, RJ-3 and RJ-4) individually, and subsequently, due to the small sample size, we analyzed the three as one. Gene diversity for each population, total heterozygosity (*Ht*) and the number of polymorphic loci *(S)* at 5% level were assessed using Arlequin (version 3.11, Excoffier *et al.*, 2005). Gene diversity was estimated, assuming independent nuclear loci and Hardy-Weinberg equilibrium.

We also calculated an additional measure of population divergence, “the frequency-down-weighted marker values” (DW), as described by Schönswetter and Tribsch (2005). For each population, the number of occurrences of each AFLP marker therein was divided by the number of that particular marker in the total dataset. To even out unequal sample sizes, DWs were calculated among 26 individuals within seven populations (considering the three Teresópolis populations as one), and among 10 randomly chosen individuals within each of the nine original populations.

#### Indirect measures of gene flow and relationships between populations 

Genetic variation within and among groups and populations was tested by a non-hierarchical analysis of molecular variance (AMOVA, Excoffier *et al.*, 1992) by using Arlequin (version 3.11, Excoffier *et al.*, 2005) with a 5% allowance for missing data. Total genetic variation was partitioned at three levels - among groups, among populations within groups and within populations. The significance of the differentiation was tested with 10.000 permutations, where P is the probability of observing a random value as large as or larger than the observed value and a confidence interval of 99.75%. Correlation among the nine populations studied was assessed through clustering analysis based on Nei's unbiased genetic distances (Nei, 1978) and the UPGMA algorithm with the use of Tools for Population Genetic Analysis (TFPGA) software (Miller, 1997). Bootstrap was calculated after 10,000 replicates.

#### Population genetic structure analysis 

Bayesian analysis with Structure Software (version 2.2, Pritchard *et al.*, 2000) was used so as to investigate population structure. Our goal was to determine the most likely number of populations (*K*). Analyses were carried out based on non-admixture and allele frequency correlated models, with 50.000 Markov Chain Monte Carlo (MCMC) steps and 10.000 burn-in periods. All the possible models for *A. angustifolia* from *K* = 2 to *K* = 12 were tried, and twelve replicates were run for each *K*. The most favourable *K* was chosen according to that suggested by Evanno *et al.* (2005).

## Results

###  Genetic diversity

The six primer-enzyme combinations used in this work yielded a total of 673 unambiguously scoreable fragments. The percentage of polymorphic loci for each primer-enzyme combination was higher in the southern populations and lower in populations RJ-2, RJ-3 and RJ-4. The highest percentage was found with PEC *Eco*RI-CG/*Mse*I-GCAC in PR (98.85%) and the lowest with PEC *Eco*RI-CT/*Mse*I-GACC in RJ-2 (30.92%). On following the same pattern, the percentage of polymorphic loci for all six primer-enzyme combinations, when considered together, yielded higher values in the southern populations and lower ones in southeastern populations RJ-2, RJ-3 and RJ-4 (Table 2). As can be seen in Table 3, population RS-1 presented the highest number of polymorphic loci (603), followed by that of PR (601). The MG population presented the lowest number of polymorphic loci (401). The estimates of gene diversity for all loci in each of the populations ranged from 0.16 to 0.27 (Table 3). The most diverse populations analysed were RS-1 and PR (0.27). On the other hand, populations RJ-2, RJ-3, RJ-4 (considered individually), RJ^#^ (RJ-2, RJ-3 and RJ-4 considered as one, with 26 randomly chosen individuals), together with the MG population, presented the lowest estimates of gene diversity (0.15-0.17). The total set of nine populations used in this analysis yielded a value for total heterozygozity of 0.27. When 26 individual were considered per population, the “frequency-down-weighted marker values” (DW) ranged from 82.54 (RS-3) to 97.51 (RJ^#^) (mean 91.9, SD = 5.37). On considering 10 randomly chosen individuals per population, DW ranged from 67.31 in population RS-2 to 84.00 in population RJ^#^ (mean 75.7, SD = 4,72; Table 3).

###  Indirect measures of gene flow and relationships between populations

As expected, the genetic distance (Nei, 1978) was the highest between RS-1 and MG (mean = 0.076), which are approximately 1034 km apart, and the lowest between populations RS-1 and RS-2 (mean = 0.006) (Table 4). In spite of their geographical proximity, the outstanding point in this analysis was RJ-1 being more distant from the other four populations from southeastern Brazil (RJ-2, RJ-3, RJ-4 and MG) than from the southern populations.

**Figure 2:**
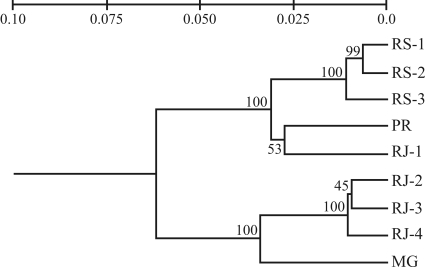
UPGMA cluster analysis using Nei's unbiased genetic distance among nine *A. angustifolia* populations. Bootstrap values after 10.000 permutations are signalled at each node.

The UPGMA dendrogram generated from AFLP data (Figure 2) was supported by high bootstrap values, thereby implying the high reliability of the pattern found. The dendrogram seems to be divided into two highly supported clusters, both of which consisting of the two regions analysed in this work - south (descendants of continuous forests) and southeast (relict populations). Nevertheless, RJ-1, which is located in the Brazilian southeast, clustered with populations from southern Brazil.

Genetic structure was assessed by using AMOVA. Four hypotheses were tested. In the first, all the nine populations were considered as being just one large group. In the second, two groups were taken into consideration, one consisting of the populations RS-1, RS-2, RS-3 and PR, and the other RJ-1, RJ-2, RJ-3, RJ-4 and MG. The third hypothesis also considered the same two groups, with the exception of RJ-1, this being included in the southern group. In the fourth, there were five different populations, corresponding to the five genetic clusters identified from Bayesian analysis when using Structure Software (Pritchard *et al.*, 2000). The first hypothesis tested yielded higher genetic variation among individuals within populations (81%). However, on contemplating the hypothesis of two groups, a significant proportion of molecular variance was assigned to differences among groups (10% and, 15%) and among populations within groups (12% and 9%). The last hypothesis was based on the results from Bayesian clustering. On identifying individuals belonging to each “genetic cluster”, independent of their original location, five different populations were formed. The differences in total genetic variation partitioned into two levels (among populations and within populations) were tested According to the analysis, there was a higher variation within (80%) and a lower one among populations (19%) All values were highly significant (p < 0.001) (Table 5).

###  Population genetic structure analysis in *A. angustifolia*

According to the approach suggested by Evanno *et al.* (2005), a population structure at only one level (*K* = 5) was indicated from Bayesian analysis The average non-admixture proportion for each population of *A. angustifolia* among the five different clusters found by means of this analysis appears in Table 6. Cluster 1 is mainly formed by individuals from the PR population. The second cluster is solely composed of individuals from populations RJ-2, RJ-3 and RJ-4. These three populations are geographically close to one another. The same pattern was observed with cluster 3, formed by populations RS-1, RS-2 and RS-3. Cluster 4 is mostly composed of the RJ-1 population, whereas cluster 5 is only formed by individuals from the MG population.

## Discussion

###  Genetic diversity and forest fragmentation

Nowadays, habitat loss and fragmentation brought about by anthropogenic activities and environmental deterioration, with the consequential breaking up of large, continuous populations into small and isolated ones, have become the subject of major concern to conservation geneticists. Populations in fragmented habitats are considered more vulnerable to demographic, environmental and genetic stochasticity, thereby facing a higher risk of local extinction (Boyce, 1992; Tilman *et al.*, 1994, Lande, 1999). In this work, nine natural populations of *A. angustifolia* were investigated in order to define to what extent forest fragmentation and natural isolation can interfere in genetic diversity. As a result, accumulated data indicate higher genetic diversity in remnant populations of *A. angustifolia* in continuous forests in southern Brazil than in, isolated, relict populations in the southeast (Tables 2  and 3). Previous studies with the species also indicated higher diversity levels in continuous forest populations compared to fragmented populations in the south itself (Auler *et al.*, 2002; Medri *et al.*, 2003; Stefenon *et al.*, 2007). The decrease in genetic diversity may occur due to a prolonged period following habitat isolation, or at short-term with the present habitat fragmentation (McDonald and Hamrick, 1996). Nevertheless, *A. angustifolia* populations from southern and southeastern Brazil underwent a similar level of exploitation during the last century. Therefore, recent deforestation could not be the main cause of the lower levels of genetic diversity in southeastern populations.

Nowadays, araucaria populations in southeastern Brazil are to be found as small isolated patches, normally restricted to higher altitudes on mountaintops (Mattos, 1994; Carvalho, 2003), thereby making gene flow difficult among populations. Pollen dispersal distance and the fine-scale genetic structure of *A. angustifolia* were analyzed by Bittencourt and Sebbenn (2008). Within the studied area, *A. angustifolia* manifested a spatial genetic structure at distances up to 75 m. Based on paternity analysis, the calculated average pollination distance was 102 m, and the effective number of pollen donors was 6.4 males, corresponding to an effective pollination neighborhood area of 2.1 ha. The authors concluded that these results imply long-distance pollen dispersion inside continuous Araucaria forests. Neertheless, the highest proportion occured at short-distances, thereby producing bi-parental and correlated mating as well as reducing variance effective size (Bittencourt and Sebbenn, 2008).

Taking into consideration the characteristics of species, populations and palynological records since the last glacial period, our results indicate that araucaria forests from Rio de Janeiro and Minas Gerais States have been isolated for thousands of years, with scarce mutual gene flow. Pollen records from Rio Grande do Sul (Behling *et al.* 2004), Santa Catarina (Behling, 1995) and Paraná (Behling 1997a), give evidence that *A. angustifolia* persisted in highland valleys during the late Pleistocene. The first wide expansion of *A. angustifolia* to the highlands in these studied regions may have occurred around 1.000 to 1.500 C^14^ yr BP, when climatic conditions became more humid, with the absence of a marked dry season. Pollen records from Morro de Itapeva (São Paulo State, Southeastern Brazil) indicate warmer but still dry weather until around 2600 C^14^ yr BP. Under these conditions, *A. angustifolia* possibly survived in humid refugia. This dry period was followed by a more appropriate climate, suitable for the expansion of *A. angustifolia* to the highlands (Behling, 1997b). Nevertheless, the expansion of *A. angustifolia* to the southeastern highlands was probably less intense than in southern Brazil, possibly since the southeastern region had gone through relatively dry periods that delayed a recovery in population size (Behling, 2002). Therefore, in southern Brazil genetic signatures of population size reduction were lost, whereas these signatures persisted in southeastern populations (Stefenon *et al.*, 2008a). Our estimates of genetic diversity indexes and DW values give support to this idea. As can be seen in table 3, populations in southeastern Brazil presented the highest DW levels. The value of DW is expected to be high in long-term isolated populations where rare markers should accumulate due to mutations (Schönswetter and Tribsch, 2005). Thus, our results indicate long-standing isolation and rare gene flow between southern and southeastern regions and among southeastern populations themselves. In addition, it is possible that southeastern populations have gone through generations of the random effects of genetic drift. Bottleneck or founder effect events might also have led to a drop in population gene diversity. A previous study with araucaria populations from south and southeast Brazil described signatures of bottlenecks in all the southeastern populations analyzed, but in only three out of the 13 southern populations (Stefenon *et al.*, 2008a). Therefore, it is possible to assume that the event may have occurred within the five southeastern populations of this research, although still further study is necessary to confirm this. Bottleneck effects could be intensified as a result of anthropogenic activities such as selective logging. Selective logging is probably another cause of the reduction in population size, thereby leading to a loss of rare alleles, a decrease in heterozigosity and increased population-inbreeding in araucaria forests. Nonetheless, even after the drastic exploitation in the last century, south Brazilian *A. angustifolia* populations still preserve their genetic diversity (Table 3). This is possibly due to forest fragmentation being recent and *A. angustifolia* a long-lived species that can live for 500 years. Due to the species high quality timber (Carvalho, 2003) and the importance of araucaria seeds as a source of nutrients for several human populations (Reitz *et al.*, 1988), the transport of its seeds by natives or other more recent human populations cannot be ignored. Thus, present-day *A. angustifolia* distribution and genetic features may have been influenced by human interference. However, the observed reduction in genetic diversity in southeastern populations and the genetic differentiation between southern and southeastern populations are probably due, in the first place, to bottleneck events since the last glacial period, followed by a genetic drift, rather than to human exploitation (Lowe *et al.*, 2004; Frankham *et al.* 2005).

###  Relationships between populations

Hamrick *et al.* (1992) pointed out the important role that a species geographical distribution and evolutionary history can play in determining the manner in which genetic diversity is distributed. The genetic distances in populations revealed an expected differentiation among southern and southeastern populations, with the exception of RJ-1 (Table 4, Figure 2). This high differentiation among southern and southeastern araucaria populations was also verified with the use of isoenzymic loci (Sousa *et al.*, 2004) and microsatellite loci (Stefenon *et al.*, 2007). In general, our results show that genetic distances among southern populations (RS-1, RS-2, RS-3 and PR) are lower than among southeastern populations (RJ-1, RJ-2, RJ-3, RJ-4 and MG). For instance, populations RS-1,2,3 and PR are about 419 km apart and their genetic distances vary from 0.0064 to 0.0312. On the other hand, populations RJ-1,2,3 and 4 are approximately 204 km apart and their genetic distances vary from 0.0096 to 0.0606 (Table 4).

Prior to recent human interference, populations from south Brazil formed part of a continuous forest, with an expected and considerable gene flow, this being mainly responsible for diminishing their genetic distance. As araucaria is a long-lived species, it is possible that we are still observing the conditions of populations before human influence. The expected differentiation among regions can be explained by the behavior of araucaria forests during the glacial and inter-glacial periods. According to palynological data, during colder and drier periods (from about 35,000 to 17,000 ^14^C years before the present) it is supposed that araucaria forests were restricted to protected valleys and wetter coastal slopes, besides bordering rivers and forming gallery forests (Behling, 1997b; Ledru *et al.*, 1998). However, with the advent of warmer climates, these forests retracted, remaining restricted to higher altitudes, with cooler temperatures and a regular rainfall. (Behling, 1995; Kershaw and McGlone, 1995; Ledru *et al.*, 1996). Therefore, since the last glacial period (about 18,000 to 15,000 years before the present; Brewer *et al.*, 2002), the southeastern araucaria populations have always formed small, normally isolated patches on mountaintops (Ledru *et al.*, 1996; Behling, 1998), with rare mutual gene flow. Thus, we can possibly assume that these small populations have undergone two genetic outcomes, genetic drift and inbreeding. Genetic drift is responsible for decreasing variation within populations (loss of alleles and heterozigosity) and increasing differentiation, whereas, inbreeding plays an important role by increasing homozygosity in small populations (Ellstrand and Elam, 1993). Both effects, added to the lack of gene flow due to natural isolation, possibly increased the genetic distance even among populations of southeastern Brazil.

The four hypotheses tested by AMOVA revealed that, despite fragmentation and natural isolation, the greater part of variation still resides within populations (81%, 77%, 74% and 80%, Table 5). These results were expected for species with an *A. angustifolia*-like behaviour. According to Loveless and Hamrick (1984), anemochoric, allogamic and long-lived perennial species normally present greater molecular variance within the population itself than among populations. Furthermore, the values arrived at in this work are close to those found in other studies on araucaria (Mazza and Bittencourt, 2000; Stefenon *et al.*, 2007) and other members of the Araucariaceae family (Bekessy *et al.*, 2002; Pye and Gadek, 2004). The second and third hypotheses tested by AMOVA indicate that when the RJ-1 population was included in the southern group, the variation among groups increased. Therefore, we can suggest that this specific population is in fact more related to the southern populations and was responsible for diminishing the differences among groups. These results corroborate genetic distance (Table 4) and UPGMA dendrogram findings (Figure 2). When the fourth hypothesis was tested (five “genetic clusters” were considered based on Bayesian analysis clustering), we also noticed the highest variation within clusters (80%) and the lowest variation among clusters (20%). Based on this result, it is possible to assume that we were dealing with five different genetic groups instead of just south-southeastern regional differentiation.

###  Populations genetic structure analysis in *A. angustifolia*

The Bayesian analysis provided insights regarding the genetic structure of *A. angustifolia* populations. First, it was possible to define an exclusive cluster for the MG population (cluster 5, Table 6) which is in agreement with its present condition. It is possible that, due to the population size and its complete isolation, it has been undergoing successive stochastic events, such as genetic drift, a reduction in genetic diversity and increasing genetic divergence among populations. In the second place, it disclosed one cluster for the three RS populations (cluster 3, Table 6) and another for the RJ-2, RJ-3 and RJ-4 (cluster 2, Table 6). Hence, we can possibly assume that in this study we were working with five genetic clusters instead of nine populations, as was predicted by their geographic locations. Third, this analysis resulted in one cluster essentially composed of individuals from population RJ-1 and with few individuals from the RS and PR populations (cluster 4, Table 6). This corroborates the genetic distance and UPGMA dendrogram findings. One genetic cluster (cluster 1, Table 6), basically composed of individuals from the PR population with some individuals from others, was also identified from Bayesian analysis. Thus, it is possible to suppose an ancient gene flow among these populations, mainly by means of pollen, the main factor responsible for long-range dispersal in *A. angustifolia* (Bittencourt and Sebbenn, 2007). It is also possible that at some time, a stepping-stone pollen-flow may have contributed to diminishing differentiation among populations (Stefenon *et al.*, 2008b). However, selective logging of *A. angustifolia* forests in south Brazil, thereby reducing its area of natural occurrence, together with the probable isolation of southeastern populations on mountaintops, especially after the last glacial period, may have made gene-flow among populations difficult. According to Frankham *et al.*, 2005, one indicator of the genetic impact of forest fragmentation and the consequent isolation is related to gene flow between populations. Forest fragmentation leads to two main events, the decrease in habitat-area and the appearance of small-sized isolated patches resulting in genetic drift (Freeland, 2005).

###  Fragmentation and implication for conservation

Genetic drift and inbreeding may influence small isolated plant populations by changing patterns of gene diversity and fitness. Both effects have implications for conservation. It is known that the loss of genetic variation may decrease the potential to persist in the face of biotic and abiotic environmental changes (Soulé, 1980; Simberloff, 1988). It may also alter the ability of a population to endure short-term challenges, such as pathogens and herbivores (Huenneke, 1991). In this work, we learned that the genetic diversity of small, isolated populations of *A. angustifolia* in southeastern Brazil has been decreasing, mainly due to a series of events that have been corroding heterozygosity and increasing allele loss, which, in the near future could lead to extinction. Thus, conservation strategies for remnant populations of araucaria, particularly those in southeast Brazil, and that take into account the genetic distinctness of each population, are of extreme importance. Some populations of the species are already located in National Parks (Parque Nacional do Itatiaia, Parque Estadual dos Três Picos, among others) and thus are protected by Brazilian law, but there are still other relict populations left that need to be further studied and protected.

## Figures and Tables

**Table 1:** Characteristics of the sampled area, number of individuals sampled and sampling material

	Itatiaia	Teresópolis				São Francisco de Paula			Mangueirinha	Liberdade
	RJ-1	RJ-2	RJ-3	RJ-4		RS-1	RS-2	RS-3	PR	MG
Sample size	26	10	12	12		26	26	26	26	26
Sample material	Needle	Cambium	Cambium	Cambium		Needle				
Latitude/longitude	22° 24' S/44° 50' W	22° 15' S/42° 37' W				29° 30' S/50° 10' W			25° 56' S/52° 10' W	22° 01' S/44° 19' W
Altitude (m)	1000	1060	800	785		1156				
Climate (Köppen)	Mesothermic humid (Cfb)	Humid Subtropical (Cfa)				Mesothermic humid (Cfb)			Mesothermic humid (Cfb)	Mesothermic and Subtropical humid (Cfa and Cfb)
Mean annual T (ºC)	11.4		17				15		19	19.1
Annual precipitation (mm)	2400		1671				2252		1900	1568

**Table 2:** Percentage of polymorphic loci for each primer-enzyme combination (PEC).

	RJ- 1 (n = 26)	RJ-2 (n = 10)	RJ-3 (n = 12)	RJ-4 (n = 12)	RS-1 (n = 26)	RS-2 (n = 26)	RS-3 (n = 26)	PR (n = 26)	MG (n = 26)
CA/GACC	78.78%	40.15%	56.81%	49.24%	89.39%	81.81%	71.96%	78.03%	40.90%
CA/GCAC	88.38%	46.45%	50.32%	45.16%	97.41%	96.83%	94.19%	84.51%	45.80%
CC/GACC	66.03%	43.39%	50.00%	40.56%	74.52%	66.98%	64.15%	88.67%	74.52%
CG/GACC	72.64%	51.88%	56.60%	62.26%	88.67%	84.90%	79.24%	94.33%	54.71%
CG/GCAC	65.51%	51.72%	49.42%	50.57%	93.10%	89.65%	96.55%	98.85%	75.86%
CT/GACC	65.97%	30.92%	40.20%	36.08%	84.53%	72.16%	89.79%	89.69%	73.19%

Total	73.69%	46.21%	53.34%	47.84%	89.59%	83.50%	79.34%	89.30%	59.58%

The percentages for each combination was calculated based on 99% criteria.

**Table 3:** Genetic diversity indexes for the nine *Araucaria angustifolia* populations analyzed.

Populations	N	S	Frag_priv._	*H*	DW (n = 26)	DW (n = 10)
RJ-1	26	496	7	0.20 ± 0.18*	95.00	74.73
RJ-2	10	311	0	0,16 ± 0.19*	-	74.62
RJ-3	12	359	0	0,17 ± 0.19*	-	82.11
RJ-4	12	322	0	0,15 ± 0.19*	-	74.69
RJ-2,3,4^#^	26	435	6	0.16 ± 0.08*	97.51	84.00
RS-1	26	603	2	0.27 ± 0.17*	87.42	71.00
RS-2	26	562	2	0.26 ± 0.18*	82.54	67.31
RS-3	26	534	4	0.23 ± 0.18*	90.39	74.69
PR	26	601	8	0.27 ± 0.17*	94.87	77.68
MG	26	401	5	0.16 ± 0.18*	95.56	77.16
Mean	21.1	465.6	3.4		91.90	75.70
Overall	190	673		0.27 ± 0.26*		

n = sample size; S = number of polymorphic loci; Frag_priv_. = number of private fragments; *H* = gene diversity; * = standart deviation (p < 0.05); ^#^ = populations RJ-2, RJ-3 and RJ-4 considered as one.

**Table 4:** Unbiased Nei genetic distances (Nei, 1978) among all the nine populations (below diagonal) and geographic distance between populations (km) (above diagonal).

	RJ-1	RJ-2	RJ-3	RJ-4	RS-1	RS-2	RS-3	PR	MG
RJ-1	—	204	204	204	978	978	978	859	56
RJ-2	0.0580	—	2	2.5	1127	1127	1127	1052	175
RJ-3	0.0474	0.0096	—	2.5	1126	1126	1126	1052	175
RJ-4	0.0606	0.0101	0.0108	—	1126	1126	1126	1052	175
RS-1	0.0291	0.0720	0.0566	0.0699	—	1	1	419	1034
RS-2	0.0362	0.0796	0.0666	0.0754	0.0064	—	1	419	1034
RS-3	0.0381	0.0741	0.0573	0.0681	0.0125	0.0092	—	419	1034
PR	0.0274	0.0446	0.0293	0.0461	0.0238	0.0312	0.0278	—	909
MG	0.0695	0.0359	0.0329	0.0330	0.0730	0.0767	0.0706	0.0403	—

**Table 5:** Analysis of molecular variance (AMOVA) for four non-hierarchical models.

Hypothesis	Source of variation	d.f.	Variance components	Variation
I	Among populations	8	17.58924	19.01
	Within populations	181	74.91766	80.99

II	Among groups (S x SE)	1	10.38166	10.70
	Among populations within groups	7	11.72282	12.08
	Within populations	181	74.91766	77.22

III	Among groups (S plus RJ-1 x SE)	1	15.95329	15.86
	Among populations within groups	7	9.72750	9.67
	Within populations	181	74.91766	74.47

IV	Among “genetic clusters”	4	18.44308	19.53
	Within “genetic clusters”		75.99819	80.47

After 10000 replicates p < 0.001, confidence interval: 99,75%.

**Table 6:** Average non-admixture proportion for each sampled population of *Araucaria angustifolia* among each of the five genetic clusters inferred from Bayesian analysis.

	Clusters				
Populations	1	2	3	4	5
RJ-1	0.077	0.000	0.000	0.923	0.000
RJ-2	0.200	0.800	0.000	0.000	0.000
RJ-3	0.268	0.566	0.000	0.167	0.000
RJ-4	0.167	0.833	0.000	0.000	0.000
RS-1	0.269	0.000	0.500	0.231	0.000
RS-2	0.116	0.000	0.654	0.231	0.000
RS-3	0.115	0.000	0.731	0.154	0.000
PR	0.755	0.000	0.000	0.245	0.000
MG	0.039	0.000	0.000	0.000	0.961
